# Eye movements reflect and shape strategies in fraction comparison

**DOI:** 10.1080/17470218.2015.1046464

**Published:** 2015-06-03

**Authors:** Anja Ischebeck, Marina Weilharter, Christof Körner

**Affiliations:** ^a^ Department of Psychology, University of Graz, Graz, Austria

**Keywords:** Fraction comparison, Number processing, Eye movements

## Abstract

The comparison of fractions is a difficult task that can often be facilitated by separately comparing components (numerators and denominators) of the fractions—that is, by applying so-called component-based strategies. The usefulness of such strategies depends on the type of fraction pair to be compared. We investigated the temporal organization and the flexibility of strategy deployment in fraction comparison by evaluating sequences of eye movements in 20 young adults. We found that component-based strategies could account for the response times and the overall number of fixations observed for the different fraction pairs. The analysis of eye movement sequences showed that the initial eye movements in a trial were characterized by stereotypical scanning patterns indicative of an exploratory phase that served to establish the kind of fraction pair presented. Eye movements that followed this phase adapted to the particular type of fraction pair and indicated the deployment of specific comparison strategies. These results demonstrate that participants employ eye movements systematically to support strategy use in fraction comparison. Participants showed a remarkable flexibility to adapt to the most efficient strategy on a trial-by-trial basis. Our results confirm the value of eye movement measurements in the exploration of strategic adaptation in complex tasks.

Fractions and proportions accompany us in daily life. For example, during the football World Cup 2014 we could assess the odds of our favourite team by comparing goal ratios (goals scored divided by goals conceded) accumulated across previous games. Processing fractions and proportions requires an assessment of the relation between two quantities. Six-month-old infants (McCrink & Wynn, 2007), apes (Sayers & Menzel, 2012), and monkeys (Vallentin & Nieder, 2008, 2010; for a review, see Jacob, Vallentin, & Nieder, 2012) were shown to process proportions to some extent. However, the abstract concept of fractions seems to be difficult to grasp and prone to errors, with students having a hard time acquiring and calculating with fractions (Mack, 1995; Meert, Grégoire, Seron, & Noel, 2013; Sprute & Temple, 2011; for reviews, see Ni & Zhou, 2005; Siegler, Fazio, Bailey, & Zhou, 2013; Siegler, Thompson, & Schneider, 2011).

Comparing two fractions is not an easy task even for adults (Schneider & Siegler, 2010). For specific fraction comparisons, however, strategies can be used to simplify the task. For example, a fraction pair such as 3/5 and 4/5 has equal denominators and can be compared on the basis of the numerators alone. Similarly, 4/5 and 4/7 can be compared on the basis of the denominators alone. Even in fraction pairs with different denominators and numerators, separate comparisons of numerators and denominators can lead to the correct comparison result. For example, in a fraction pair like 2/7 and 5/3, the two separate comparisons of the numerators (2–5) and the denominators (7–3) yield the right fraction as the larger one. When both comparisons lead to different responses, however, the fraction pair can no longer be solved by a comparison of its components alone. Strategies based on comparisons between numerators and denominators have often been described (Bonato, Fabbri, Umiltà, & Zorzi, 2007; Faulkenberry & Pierce, 2011; Ganor-Stern, Karsik-Rivkin, & Tzelgov, 2011; Ischebeck, Schocke, & Delazer, 2009; Meert, Grégoire, & Noël, 2009, 2010). These strategies are in common referred to as component-based strategies.

Strategies other than component-based strategies are also used in fraction comparison. For example, fractions are compared on the basis of their numerical value (DeWolf, Grounds, Bassok, & Holyoak, 2014; Faulkenberry & Pierce, 2011; Gabriel, Szusc, & Content, 2013; Ganor-Stern, 2012; Ganor-Stern et al., 2011; Ischebeck et al., 2009; Jacob & Nieder, 2009; Kallai & Tzelgov, 2009; Meert et al., 2009, 2010; Schneider & Siegler, 2010). It has therefore been proposed that a hybrid strategy is used by participants: They make use of component-based strategies when applicable but also compare the numerical values of fractions (Meert et al., 2009, 2010). In the present article, we focused on component-based strategies and investigated the flexibility with which these strategies can be employed depending on the type of fractions to be compared.

We compared four different types of fraction pairs (see also Ischebeck et al., 2009): fraction pairs with the same denominators (SD), pairs with the same numerators (SN), and mixed pairs, where denominators and numerators were different. There were two kinds of mixed pairs, congruent (CO, solvable by component-based strategies) and incongruent pairs (IC). Consistent with a component-based strategy, Ischebeck et al. (2009) observed faster response times and lower error rates in the SD condition, followed by the SN, CO, and IC conditions. SD fraction pairs were easier than the SN pairs, because here the larger of the two numerators indicates the larger fraction, whereas the value of the denominators is related inversely to the value of the two fractions. In turn, CO pairs were more complex than the SN and SD pairs because both numerators and the denominators have to be compared. To IC pairs, a component-based processing strategy can no longer be successfully applied. Studies have shown that response times and error rates mirror these expected differences in task difficulty (Huber, Moeller, & Nuerk, 2014; Ischebeck et al., 2009; Meert et al., 2009, 2010).

The choice and application of an appropriate component-based strategy depend on the properties of the type of fraction pair at hand. When different fraction pair types are presented, it is important to note that a suitable strategy can only be applied after the type of fraction pair has been determined. It therefore makes sense to break down the process of fraction comparison into (at least) two phases, a first exploratory phase and a comparison phase. The exploratory phase serves to determine the type of fraction pair, while in the comparison phase a strategy is applied, and the actual fraction comparison is executed.

A means to investigate different phases in the processing of fractions is the measurement of eye movements. Eye movement recordings yield more information than response times. For example, the number of fixations can inform about the amount of processing devoted to specific fraction components (Huber et al., 2014). Furthermore, they allow tracking of the processing within a trial on a moment-by-moment basis. The temporal sequence of fixations (the so-called scan path) might be particularly informative with regard to viewing and comparison strategy. It can be expected that participants’ eye movements follow and reflect their choice of a fraction comparison strategy.

When a fraction pair is first presented, the initial eye movements may serve to explore the fractions’ components. As participants do not know at this point what fraction pair is presented, eye movement patterns might be rather unspecific with regard to the type of fraction pair. Once the type of fraction is identified, however, it can be assumed that eye movements adapt to the type of fraction pair—for example, if a component-based strategy can be applied successfully. Whereas eye movements in the exploratory phase are thought to be more stereotypical—that is, they might follow fixed specified patterns—we expect that eye movements become ever more specific and indicative for a specific comparison strategy during the course of fraction processing. In this article, it is our goal to investigate systematic eye movement patterns in these two phases and relate them to each other.

To the extent of our knowledge, eye movements have been used only once before to explore fraction comparison (Huber et al., 2014). In this study, eye movements were found to indicate the application of a component-based strategy. When the two fractions’ denominators (numerators) were identical, numerators (denominators) were fixated relatively more often. This indicated that participants compared numerators and denominators separately in these conditions (component-based strategy), especially when fraction pairs of the same type were presented in blocks, compared to a random presentation. The authors also presented fraction pairs where denominators and numerators differed and observed more fixations in this more difficult condition. The authors also observed that, overall, denominators were fixated more often than numerators, possibly indicating an increased difficulty in processing the inverse relationship of the magnitude of the denominator to the magnitude of the whole fraction.

In the present experiment, we analyse response times and error rates as well as the number of fixations as a measure of task difficulty. Our main analyses, however, focus on the decomposition of scan paths into an exploratory phase and a comparison phase. We first analyse the initial exploratory four fixations. We hypothesize that these fixations primarily serve to determine the type of fraction pair at hand. Therefore, these fixations are expected to be more stereotypical—that is, following mainly a few fixed fixation patterns that are unspecific for the type of fraction pair to be compared. These patterns could, for example, be determined by reading direction (from left to right). Furthermore, it is possible that already at this early stage specific viewing patterns may have an impact on overall processing. For example, given the important role of the denominators, we expect that exploratory viewing patterns that include denominator–denominator transitions might speed up overall response time in the fraction comparison task.

We then analyse sequences of three consecutive fixations (scan path triplets) for each fraction pair, following the initial four fixations. If SD fraction pairs are to be compared, it is expected that numerators are fixated in alternation and more often. If SN fraction pairs are compared, it is expected that denominators are fixated alternatingly and more often. In mixed pairs trials (CO and IC), it is expected that the components of either the right or the left fraction are fixated alternatingly and more often. This viewing pattern could be taken to indicate that participants estimate the value of a fraction. Last, we examine what components are inspected with the final two fixations. For these fixation pairs we expect a very systematic viewing pattern, depending on the type of fraction pair. Whereas the four initial fixations are not expected to depend much on the type of fraction pair presented, this dependency is expected to increase systematically for eye movement sequences later in the processing pipeline. All analyses are carried out for single-digit fractions and, as an extension, for double-digit fractions.

## Method

### Participants

Twenty young adults (14 female, mean age: 24.5 years, *SD* = 4.4), most of them psychology students, took part in this experiment. All participants had normal or corrected-to-normal vision. Psychology students received course credit for their participation; other participants took part without compensation. They had all given written informed consent. The experiment was approved by the ethics committee of the University of Graz.

### Design and stimuli

The experiment was divided into two parts. In Part 1, participants compared pairs of single-digit fractions. In Part 2, which followed Part 1, participants compared double-digit fractions. All fractions were irreducible. Unit fractions like 1/2, 1/3, 1/4, 1/5, and so on, were excluded. A total of 62 different pairs of two single-digit fractions and 32 different pairs of two double-digit fractions were created (see Table 1 for a listing of all used fraction pairs). Same denominator (SD) fraction pairs had different numerators (example: 2/7 and 5/7). Same numerator (SN) pairs had different denominators (example: 3/5 and 3/8). In all other fraction pairs, the numerators and the denominators were always different. In the congruent condition (CO), the separate comparisons of the numerators and denominators could still lead to the same response (example: 3/5 and 2/8; numerators: 3 is greater than 2 yields the left fraction as the greater fraction; denominators: 5 is smaller than 8, which also yields the left fraction as the greater fraction). In the incongruent condition (IC), the separate comparison of numerators and denominators led to different responses (example: 2/5 and 3/8; numerators: 3 is greater than 2 yields the right fraction as the greater fraction; denominators: 5 is smaller than 8, which yields the left fraction as the greater fraction).

**Table 1.** Fraction pairs and their properties as used in the experiment

Fractions	Fraction pair	Partial distance	Proper fractions	Improper fractions
Single-digit fractions	SD	1		
		3		
	SN	1		
		3		
	CO	1		
		3		
	IC	1		
		3		
Double-digit fractions	SD	n/a		
	SN	n/a		
	CO	n/a		
	IC	n/a		

*Note:* SD = same denominators; SN = same numerators; CO = congruent; IC = incongruent.

Numbers are compared faster the greater their numerical distance (distance effect, Moyer & Landauer, 1967). If fractions are compared using component-based strategies, a distance effect on the basis of the fractions’ components should be observed. We constructed the single-digit fractions with common components in such a way that the difference between the numerators or denominators (partial distance) alternated between one and three (e.g., partial distance 1: 3/7 2/7; partial distance 3: 2/7 5/7). For single-digit fractions with different components, the difference between the numerators or denominators could be one or three, with the distance between the other components (denominators or numerators) being fixed to one (e.g., partial distance 1: 2/9 3/8; partial distance 3: 2/7 3/4).The real numerical distance between the two fractions varied accordingly (see Table 2 for the mean distances, partial and numerical, for all four types of fraction pairs and for single- and double-digit fraction pairs).

**Table 2.** Average numerical and partial distance of the single-digit and double-digit fraction pairs

Fractions	Fraction pair	Numerical distance	Partial distance
Single-digit fractions	SD	0.37	0.97
	SN	0.55	0.97
	CO	0.76	1.5
	IC	0.34	1.5
Double-digit fractions	SD	0.17	1.44
	SN	0.19	1.56
	CO	0.18	1.31
	IC	0.17	2.25

*Note:* SD = same denominators; SN = same numerators; CO = congruent; IC = incongruent.

We used fractions with a numerical value greater than one (improper fractions) as well as fractions with a numerical value smaller than one (proper fractions). This choice decorrelated partial and numerical distance (single-digit fractions: *r* = .37, double-digit fractions: *r* = .28). For double-digit fractions, partial distance varied only implicitly. In all other aspects the double-digit fractions had similar properties to those of the single-digit fractions.

**Figure 1:**
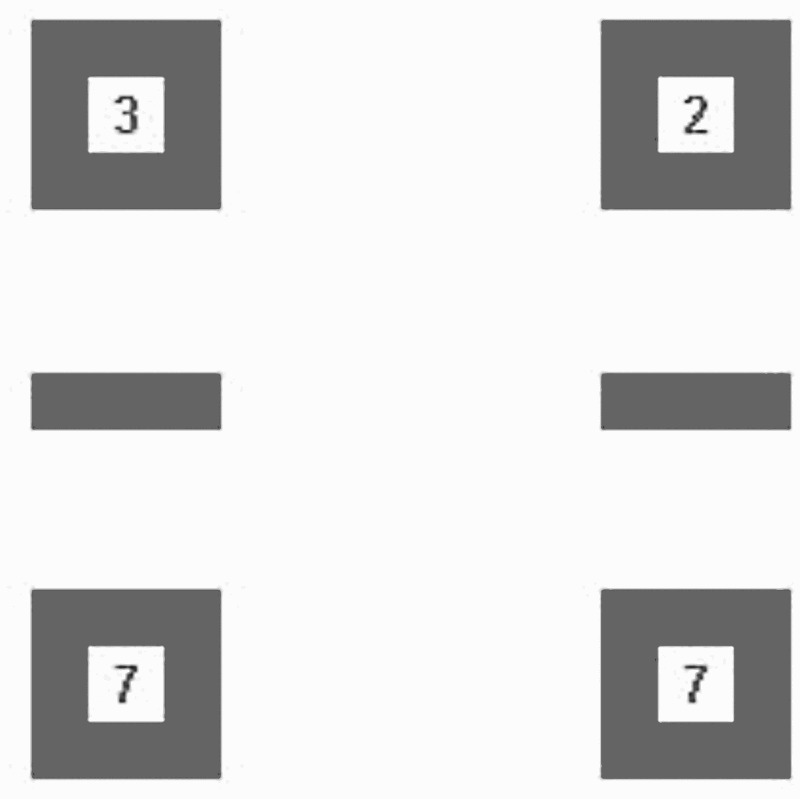
Example for a stimulus display presented in the single-digit part of the experiment. The illustration is enlarged, and the colours are reversed for readability. (See text for precise dimensions.)

Stimuli were presented in grey on a black background. Each component (numerator, denominator) of each fraction was placed at the same distance from the centre of the screen. The distance between the fractions and between numerator and denominator of each fraction was 4.77° visual angle at a viewing distance of 63 cm. The height of a digit was 0.27° for the single-digit fractions and 0.21° for the two-digit fractions. Each digit was surrounded by a square with an edge length of 1.59° and a line thickness of 0.48°. The fraction line was 1.59° wide and 0.48° thick and was placed at equal distance between numerator and denominator. The squares surrounding the digits served two purposes. They reduced the ability to identify the digit without fixation (Bouma, 1970) and provided a clear target for the planning of the saccade. In a pilot experiment, we demonstrated that digit identification did not differ reliably from chance when fixation was more than three degrees away from such stimuli (Körner & Gilchrist, 2007). An example for the display is given in Figure 1.

All manipulations were carried out within subjects. We recorded participants’ choice of the greater fraction, the corresponding response times, and their eye movements.

### Procedure

Participants were seated in front of a monitor at a viewing distance of 63 cm. In their hands they held a two-button response box, which they operated with their thumbs. Before the start of Part 1, participants practised on 10 single-digit fraction pairs. Part 1 consisted of two blocks with 124 trials each. There was a small break between blocks. In each block, two repetitions of the 62 fraction pairs listed in Table 1 were presented in random order. In half of the trials of a block, the larger fraction was presented on the left; in the other half of trials it was presented on the right-hand side of the display. In sum, there were 248 trials in Part 1. After a break of approximately 10 minutes, participants started working on Part 2, which consisted of a single block of 128 trials. These trials resulted from four repetitions of the 32 double-digit fraction pairs listed in Table 1. In half of the trials, the larger fraction was presented on the left, and in the other half on the right side of the display.

At the beginning of each trial, a fixation point was presented in the centre of the screen. The fraction display was presented when the fixation on the fixation point was registered. Participants were instructed to press the button on the side of the greater of the two fractions. They were instructed to respond as quickly and as accurately as possible. Participants were not instructed to use a particular strategy in solving the fraction comparison problems. The fraction display was presented until the participant pressed one of two buttons on the response box. Trial-by-trial feedback was given during the practice trials to make sure the participants understood the task. No feedback was given during the experimental trials. The entire experiment lasted maximally 2 hours including breaks.

### Apparatus

Participants were seated in a darkened and acoustically insulated booth. We recorded two-dimensional eye movements using an Eye-Link II eye tracker (SR Research, Canada). This is a head-mounted system that uses two infrared cameras that monitor the eyes at a sampling rate of 500 Hz. It also uses a head movement compensation mechanism. We calibrated both eyes and recorded from the eye that produced the better spatial resolution, which was typically better than 0.30°. Displays were presented at a distance of 63 cm on a 21″ monitor with a resolution of 1152 × 864 pixels using custom-written software in C++. A chin-rest was used to minimize head movement. The velocity threshold for saccade detection was set to 35°/s; the acceleration threshold was set to 9500° s^–^². The eye tracker was calibrated before each block using a 9-point calibration procedure. A drift correction (operated by the experimenter) was performed before each trial.

### Results

In total, data from 4960 trials were collected for the single-digit fractions. After removal of trials with response times longer than 10,000 ms, there were 4752 trials (95.8%) available for analysis. For the two-digit fractions, 2560 trials were collected in total. After removal of trials with response times longer than 10,000 ms, there were 2489 trials (97.2%) available for analysis. Each fixation was assigned to the numerator or denominator component with the smallest Euclidean distance. Consecutive fixations of the same component were treated as a single fixation.

First, we report error rates, manual response times and overall number of fixations. Next, we report analyses of different subsections of the scan path of a trial.

#### Error rates, manual response times, and overall number of fixations

##### Single-digit fractions

We evaluated error rates, response times (RTs) and the overall number of fixations per trial for investigating task difficulty and for a first assessment of component-based strategies. Fixation frequencies indicate task difficulty similar to the manual responses. We expected that fraction pairs with longer RTs and higher error rates would also show a higher number of fixations. If participants used a component-based strategy, the SD condition should be the easiest (i.e., produce faster response times and fewer fixations), followed by the SN, CO, and IC conditions. Furthermore, a partial distance effect should be observed, with comparisons with a greater partial distance being easier. If fractions were compared mainly on the basis of their real value, the condition with a larger numerical distance should be easier—that is, the CO condition should be easiest, followed by the SN, SD, and IC conditions.

The overall error rate was 14.35%. Error rates were highest for the IC fraction pairs (29.2%), compared to the CO (5.6%), SN (4.1%), and SD (2.6%) pairs. There were three participants who performed at chance level with IC pairs but whose error rates were inconspicuous otherwise. In a 4 × 2 analysis of variance (ANOVA) with repeated measures and fraction pair and partial distance as factors, this was reflected in a main effect of fraction pair, *F*(3, 57) = 58.9, *p* < .001, 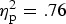. Partial distance also had an effect, with significantly more errors being made for partial distance 1 (13.2%) than for partial distance 3 (7.6%); main effect of partial distance, *F*(1, 19) = 24.2, *p* < .001, 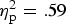. The influence of partial distance differed with respect to fraction pair, yielding a significant Partial Distance × Fraction Pair interaction, *F*(3, 57) = 21.2, *p* < .001, 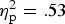. Tukey honestly significant difference (HSD) tests showed that error rates for IC fraction pairs were higher than those for all other conditions and differed between partial distance 1 and partial distance 3 (see Table 3).

**Table 3.** Mean error rates, response times, and number of fixations for single-digit and double-digit fraction pairs

Fractions	Fraction pair	Partial distance	Error rate	Response time	Number of fixations
Single-digit fractions	SD	1	3.13 (6.88)	2733 (954)	6.70 (1.33)
		3	2.26 (5.08)	2751 (877)	6.75 (1.37)
	SN	1	4.52 (6.0)	3245 (1158)	7.57 (1.85)
		3	3.75 (5.96)	3100 (1222)	7.17 (1.89)
	CO	1	6.17 (7.36)	3696 (1327)	8.58 (2.51)
		3	4.96 (5.91)	3534 (1235)	8.26 (2.38)
	IC	1	38.88 (14.87)	4187 (1720)	9.38 (3.13)
		3	19.53 (16.99)	3991 (1528)	9.01 (2.88)
Double-digit fractions	SD	n/a	2.19 (3.38)	2554 (688)	6.28 (1.12)
	SN	n/a	0.64 (1.67)	3006 (1047)	7.12 (2.03)
	CO	n/a	4.25 (8.15)	3896 (1276)	8.86 (2.62)
	IC	n/a	17.99 (17.62)	4138 (1422)	9.41 (3.00)

*Note:* Response times in ms. Standard deviations in parentheses. SD = same denominators; SN = same numerators; CO = congruent; IC = incongruent.

After the removal of incorrect trials, there were data from 4248 correct trials available for the analysis of RTs. Participants responded indeed fastest to fraction pairs from the SD condition (2742 ms), followed by the SN (3173 ms), CO (3615 ms), and IC (4089 ms) conditions. In a 4 × 2 ANOVA with repeated measures and fraction pair and partial distance as factors, this was reflected in a significant main effect of fraction pair, *F*(3, 57) = 20.45, *p* < .001, 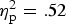. Further evidence for a component-based strategy came from the observation of a partial distance effect. Fraction pairs with partial distance 1 (3465 ms) were responded to more slowly than fraction pairs with partial distance 3 (3344 ms); main effect of partial distance, *F*(1, 19) = 5.58, *p* < .05, 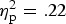. The interaction Fraction Pair × Partial Distance was not significant, *F*(3, 57) < 1, *p* = .54. The mean RTs are shown in Table 3.

To investigate whether numerical distance influenced RTs, a general linear model was calculated for RTs averaged per item. Fraction pair was entered as a categorical predictor and numerical distance as a continuous predictor. Fraction pair was a significant predictor of RT, *F*(3, 57) = 49.06, *p* < .001, as well as numerical distance, *F*(1, 57) = 16.95, *p* < .001. We also analysed the influence of numerical distance per fraction pair on RTs, by calculating a general linear model (separate slopes model). Fraction pair was entered as a categorical predictor and numerical distance as a continuous predictor. Numerical distance effects differed between fraction pairs, which was reflected in a significant interaction between fraction pair and numerical distance, *F*(4, 54) = 6.68, *p* < .001. Significant partial (*pc*) and semipartial correlations (*sc*) for numerical distance were observed for fraction pairs CO (*pc* = −.46, *sc* = −.25, *p* < .001), and IC (*pc* = −.35, *sc* = −.17, *p* < .01).

Manual RTs and overall number of fixations are typically highly correlated (Williams, Reingold, Moscovitch, & Behrmann, 1997). We therefore expected that longer trials require a higher number of fixations. Indeed, the number of fixations was smallest in the SD condition (6.73), followed by the SN condition (7.37), the CO condition (8.42), and the IC condition (9.19). In a 4 × 2 ANOVA with repeated measures and fraction pair and partial distance as factors, this was reflected in a significant main effect of fraction pair, *F*(3, 57) = 17.71, *p* < .001, 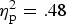. Fraction pairs with partial distance 3 (7.80) were fixated less often than fraction pairs with partial distance 1 (8.06), leading to a significant main effect of partial distance, *F*(1, 19) = 6.55, *p* < .05, 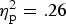. The interaction was not significant, *F*(3, 57) < 1, *p* = .41. The number of fixations is summarized in Table 3.

##### Double-digit fractions

Overall error rate was 6.27%. Error rates were entered into a one-way ANOVA with factor fraction pair (SD, SN, CO, IC). As expected, most errors were made in the IC condition, followed by the CO, SD, and SN conditions, resulting in a significant effect of fraction pair, *F*(3, 57) = 13.40, *p* < .001, 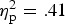.

For the analysis of RTs, data from 2333 correct trials were available after the removal of incorrect trials. Similar to the single-digit fractions, RTs for the double-digit fractions differed between fraction pairs, which was reflected in a significant effect of fraction pair, *F*(3, 57) = 30.87, *p* < .001, 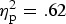. RTs were fastest in the SD condition, followed by the SN, CO, and IC conditions (see Table 3).

Similar to the one digit-fractions, fraction pair was a significant predictor of RTs for the two-digit fractions, *F*(3, 27) = 51.40, *p* < .001, but numerical distance was not, *F*(1, 27) = 16.95, *p* = .124. Numerical distance effects did not differ significantly between fraction pairs. A significant partial (*pc*) and semipartial correlation (*sc*) for numerical distance was observed in the fraction pair IC (*pc* = −.44, *sc* = −.16, *p* < .05). These results were probably due to the overall lower number of different double-digit fraction pairs (*n* = 32) than of single-digit fraction pairs (*n* = 62).

With regard to the number of fixations, a similar pattern of results was observed. There were fewer fixations in the SD condition, followed by the SN condition, the CO condition, and the IC condition. This was reflected in a significant effect of fraction pair, *F*(3, 57) = 25.20, *p* < .001, 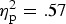 (see Table 3).

#### Scan path analysis

In this part of the Results section, we report analyses of the four initial fixations, the subsequent fixations, and the final two fixations.

##### Initial four fixations

We first consider the scan path consisting of the first four fixations. Owing to the careful construction of our displays (see above), it was impossible to identify the fractions’ components without directly fixating on them. A participant must therefore make at least four fixations (each on a different component) to determine what fractions are to be compared. Considering that four bits of information are within short-term memory limits, and assuming an efficient scanning strategy, participants can determine the type of fractions within four fixations. We therefore consider the initial four fixations exploratory. They might be stereotypical; that is, they might mainly follow fixed fixation patterns—for example, determined by reading direction. Furthermore, the initial four fixations might already determine subsequent processing and influence response times.

**Figure 2:**
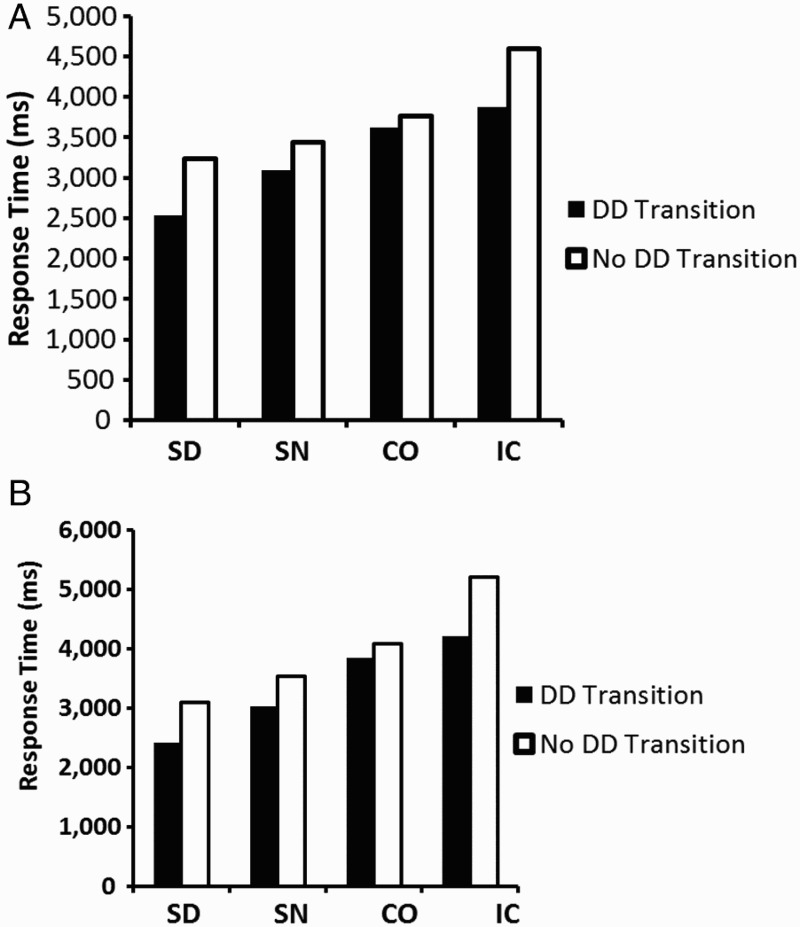
Response times for trials with a denominator–denominator transition (DD) within the first four fixations (black bars) and without such a transition (white bars), for each fraction pair, for (A) single-digit fractions, and (B) double-digit fractions. SD = same denominators; SN = same numerators; CO = congruent; IC = incongruent.

*Single-digit fractions*. The total number of fixations in a trial (i.e., the length of the scan path) averaged across all conditions was 7.93 (*SD* = 0.67, see Table 3). We analysed the initial four fixations of the scan path. There were 5.81% of trials in which participants made fewer than four fixations; these trials were not analysed. The initial four fixations may represent a “first screening” of the fractions that serves to determine what kind of fraction pair is presented and to plan a subsequent comparison strategy. In this case we would expect that each of the four components is fixated exactly once during the initial four fixations. Indeed, in 60.97% of the trials, participants fixated each of the four components once, indicating a very efficient scanning strategy. In the remainder of the trials, participants refixated components. For example, refixations of the first component with the third fixation accounted for 24.9% of the trials. Such refixations (or regressions) may be part of a general oculomotor strategy and are commonly observed in text reading (Rayner, 1998). In these trials, participants needed more than four exploratory fixations. Such fixation patterns are more complex and less straightforward to analyse. In the subset of 60.97% of the trials, where each of the four components was visited once, the most common sequence of fixations (37.31%) started with the fixation of the numerator of the left fraction (NL) and proceeded “counterclockwise”—that is, the denominator of that fraction was fixated next (DL), followed by the denominator of the right fraction (DR) and the numerator of the right fraction (NR). The second most common sequence (10.57%) was NL–DL–NR–DR. Thus, in the majority of trials, participants did inspect all components but the pattern of fixations was not the same. The most prominent sequence included a transition between the denominators (DD transition), whereas the second most prominent sequence did not. It is possible that such subtle differences in initial fixation sequences influence subsequent fraction processing. We therefore analysed whether the existence of a DD transition within the initial fixations was related to the response time of the entire trial. We expected that SN fraction pairs would benefit most from such a transition, as here only the denominators have to be compared. We compared all trials with a DD transition (61.32%) among the initial four fixations to those without, for each fraction pair. (Because one participant did not produce a SD trial without DD transition, the following analysis was based on data from 19 participants.) A 2 (transition) × 4 (fraction pair) ANOVA showed that RTs increased across fraction pairs, *F*(3, 54) = 19.62, *p* < .001, 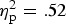 (see Figure 2A; cf. also Table 3). RTs were generally shorter in trials with DD transition (3758 ms, *SD* = 289) than in the other trials (3282 ms, *SD* = 251), *F*(1, 18) = 18.30, *p* < .001, 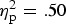. (This was a robust effect; all but two participants showed this RT advantage for DD transitions for at least three of the fraction pairs.) The interaction was also significant, *F*(3, 54) = 4.46, *p* < .01, 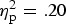. Tukey HSD tests revealed that the RTs were significantly shorter for trials with a DD transition for SN and IC fraction pairs (*p*s < .01).

*Double-digit fractions.* There were 5.40% of trials in which participants made fewer than four fixations. These trials were not analysed. In 69.74% of the trials, participants fixated each of the four components within the first four fixations. Again, the most common sequence of fixations (40.76%) was NL–DL–DR–NR. The second most common sequence (14.06%) was DL–DR–NR–NL, also involving a DD transition. There were four participants who did not produce any DD transitions for some of the fraction pairs. Therefore the following comparison is based on data of only 16 participants. Response times were faster in trials with DD transition (3375 ms, *SD* = 272) than in the other trials (3979 ms, *SD* = 346), resulting in a main effect, *F*(1, 14) = 16.90, *p* < .001, 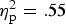 (see Figure 2B). (Again, this effect was robust; all but three of the analysed participants showed this RT advantage for DD transitions.) Response times also increased across fraction pairs, *F*(3, 42) = 14.61, *p* < .001, 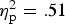. The interaction was not significant, *F*(3, 42) = 1.19, *p* = .326.

These results show that the first four fixations are rather stereotypical—that is, they mainly followed two fixed scan paths. Furthermore, they not only serve to determine what kind of fraction pair is presented, but also can be beneficial for the ensuing comparison process.

##### Scan paths following the initial fixations (triplet sequences)

We now consider the scan path from the fifth fixation onwards, considering triplets of three consecutive fixations. Triples that include the same two components X and Y—for example, X–Y–X—unambiguously indicate a comparison of these components. A single transition between X and Y might also indicate a comparison but is more ambiguous. For example, such a transition could arise as part of a longer sequence (e.g., A–X–Y–B), which may designate comparisons between components A and X, as well as Y and B, but not necessarily X and Y. We investigated whether numerators or denominators were fixated alternatingly, suggesting a comparison on the basis of the fraction's components. In the case of SD pairs, we expected that numerators are fixated alternatingly more often, whereas the opposite pattern is expected for the SN pairs.

**Figure 3 F0003:**
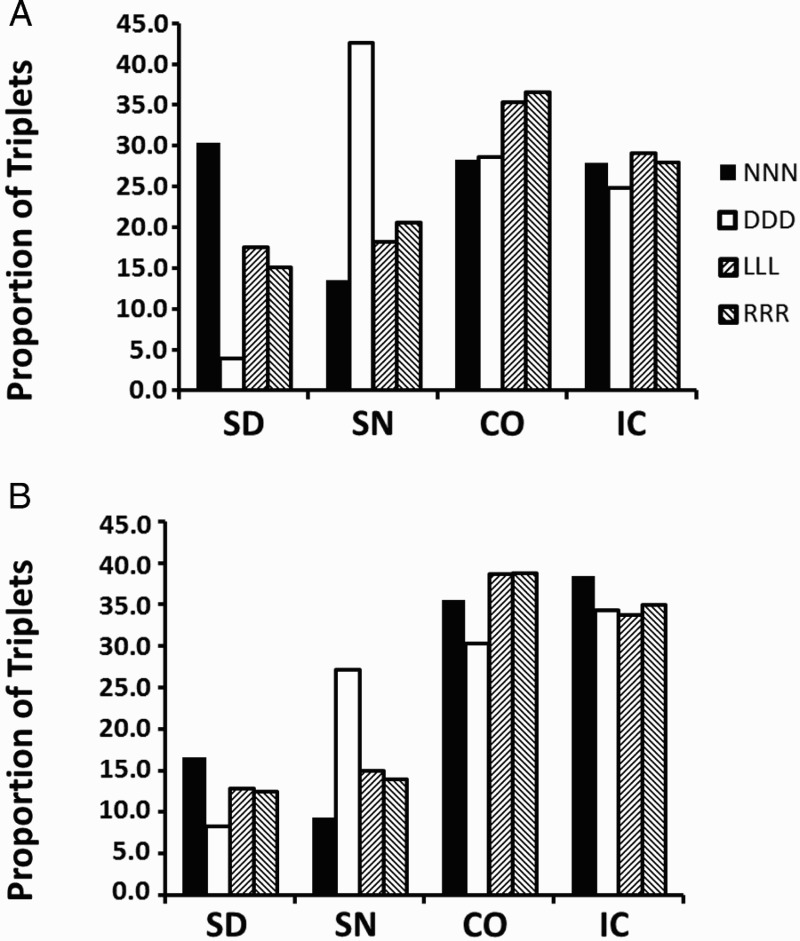
Proportion of triplet fixation sequences occurring after the initial four fixations, for each fraction pair, for (A) single-digit fractions, and (B) double-digit fractions. Note that the proportions per triplet sum up to 100% across fraction pairs. NNN = numerator triplet; DDD = denominator triplet; LLL = fraction-left triplet; RRR = fraction-right triplet; SD = same denominators; SN = same numerators; CO = congruent; IC = incongruent.

*Single-digit fractions.* Out of the original 4248 correct trials, there were 2543 (59.86%) trials in which three or more fixations occurred after the initial four fixations; these trials could be used for the triplet analysis. (This is a substantial reduction of data. In comparison, there were 74.62% of trials in which two or more fixations occurred. Such trials could have been used to analyse single transitions between two components instead of triplets. As pointed out above, single transitions are ambiguous, and the advantage of unambiguity may outweigh the reduction of data.) Within this set, we counted how often the scan path contained a fixation triplet on numerator components (NL–NR–NL, or NR–NL–NR); this was coded as NNN triplet. The same was done for denominator triplets DDD. We also coded fraction-left triplets LLL (NL–DL–NL, or DL–NL–DL) and, equivalently, fraction-right triplets RRR. There were 1783 trials (70.11%) that contained such triplets. Considering random fixation behaviour, triplets with fixations of three different components are two times more likely than triplets where saccades switch between two components. The high proportion of the specific NNN, DDD, LLL, and RRR triplets observed here highlights that participants used eye movements systematically to serve in a comparison process. For each participant, we determined the proportion of the respective fixation triplet in such a way that the proportions per triplet summed up to 100% across fraction pairs. As three participants did not produce fixation sequences in some of the conditions, their data were not included in the following analysis. The results are shown in Figure 3A. A 4 (fraction pair) × 4 (triplet) ANOVA showed that proportions differed between fraction pairs, with CO and IC fraction pairs showing a higher proportion of fixation triplets on average than SD and SN fraction pairs, *F*(3, 48) = 5.84, *p* < .001, 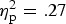. There was no main effect of triplet, *F*(3, 48) < 1. Importantly, the interaction was significant, *F*(9, 144) = 8.75, *p* < .001, 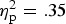. In particular, participants produced 30.27% NNN triplets when solving SD fraction pairs as opposed to only 13.53% when working on SN pairs. Conversely, there were only 3.87% DDD triplets for SD fraction pairs as opposed to 42.65% of such triplets for SN fraction pairs. Figure 3A suggests that LLL and RRR triplets did not differ for the different fraction pairs. We therefore reran the above analysis but omitted these triplets. Both main effects were only marginally significant, *F*(3, 48) = 2.56, *p* = .07 (fraction pair), and *F*(1, 16) = 19.05, *p* = .07 (triplet); as expected, the interaction was significant, *F*(3, 48) = 13.63, *p* < .01, 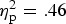. Tukey HSD tests showed that the percentage of NNN triplets was greater than the percentage of DDD triplets for SD fraction pairs, while the percentage of DDD triplets was greater than the percentage of NNN triplets for SN fraction pairs. Also, the percentage of DDD triplets for SD pairs was smaller than any other percentage, except for the NNN triplet percentage for SN pairs (*p*s < .05).

*Double-digit fractions.* Out of the original 2333 correct trials, there were 1348 (57.78%) trials in which three or more fixations occurred after the initial four fixations. Within this set, there were 876 trials (64.99%) that contained triplets of interest (NNN, DDD, LLL, RRR). The proportion of respective triplets for each of the fraction pairs is illustrated in Figure 3B. (Data from two participants who did not produce fixation sequences in some of the conditions were not included in the following analysis.) There was a main effect of fraction pair, *F*(3, 51) = 17.82, *p* < .001, 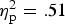, indicating that the proportion of triplets differed between fraction pairs. Figure 3B suggests that more triplets occurred for fraction pairs CO and IC than for SD and SN. There was no main effect of triplets, *F*(3, 51) = 1.59, *p* = .20, and no interaction, *F*(9, 153) = 1.13, *p* = .34. However, Figure 3B clearly shows that NNN triplets accounted for the highest proportion among SD fraction pairs (16.62%) but the lowest proportion among SN fraction pairs (9.37%), while this pattern was reversed for DDD triplets (8.26% and 27.17%, respectively). When we restricted the above analysis by including only these two kinds of triplets and fraction pairs as factors, the interaction was significant, *F*(1, 17) = 6.40, *p* < .05, 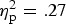, but none of the main effects, *F*(1, 17) = 3.13, *p* = .10 (fraction pair), and *F*(1, 17) = 2.91, *p* = .11 (triplet), as expected.

##### Final fixations

As a last step, we analysed the transition between the final pair of fixations of a trial before the manual response occurred. There are three classes of transitions that may occur: transitions between the same component (numerator or denominator) of different fractions, denoted as NN and DD; transitions between both components of the same fraction (left or right), denoted as LL and RR; and transitions between a numerator or denominator of one fraction and a different component of the other fraction (different components/fractions). The first class of same-component transitions is particularly informative for the application of a component-based strategy depending on the type of fraction pair.

**Figure 4:**
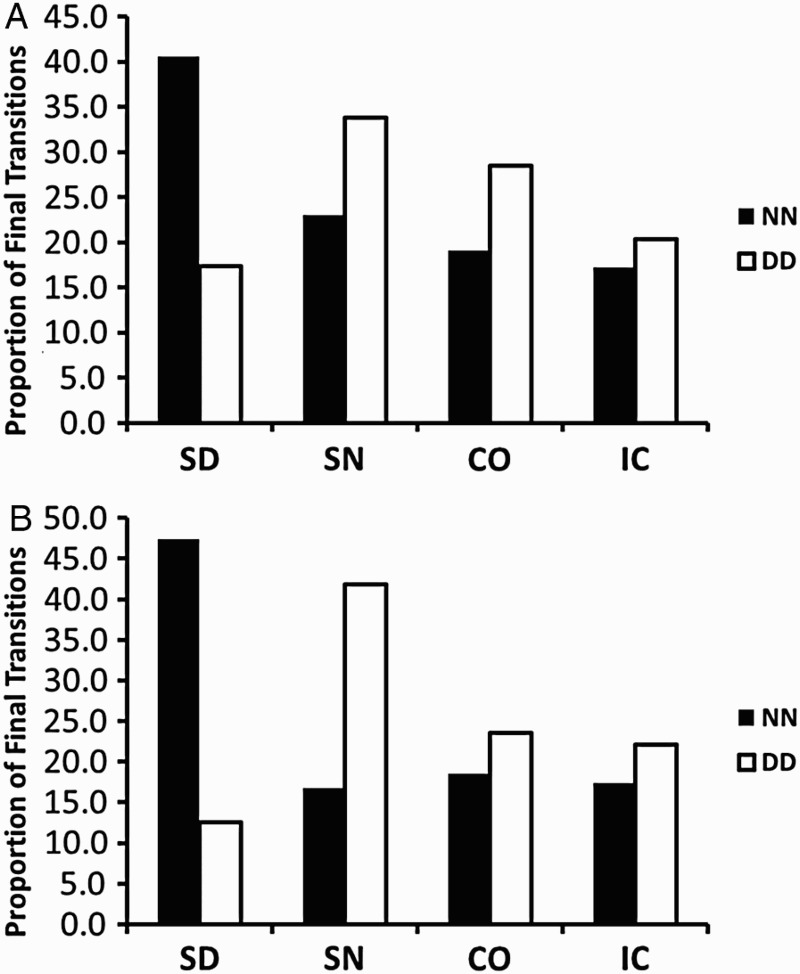
Proportion of numerator–numerator final fixation transitions NN (black bars) and denominator–denominator transitions DD (white bars), for each fraction pair, for (A) single-digit fractions, and (B) double-digit fractions. SD = same denominators; SN = same numerators; CO = congruent; IC = incongruent.

*Single-digit fractions.* Final transitions were very systematic. Overall, the same component of either fraction was fixated consecutively in 41.8% of the trials (NN: 22.64%; DD: 18.43%), or the same fraction was fixated in 47.56% of trials (LL: 19.59%; RR: 27.96%). Only 11.37% of transitions occurred for different components/fractions. In the following analysis, we determined the proportion of NN and DD fixation transitions for each kind of fraction pair in such a way that these proportions sum up to 100% across fraction pairs (see Figure 4A). NN transitions occurred most often for SD fraction pairs (40.60%), while DD transitions were more prominent for the other types of fraction pairs. This pattern was confirmed by a main effect of fraction pair, *F*(3, 57) = 9.17, *p* < .001, 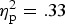, and an interaction of Fraction Pair × Final Transition, *F*(3, 57) = 10.74, *p* < .001, 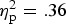. The main effect of final transition was not significant, *F*(1, 19) < 1.

*Double-digit fractions.* The final transitions for double-digit fractions were distributed even more systematically. In 55.73% of the trials the same component was fixated, the same fraction was fixated in 34.22% of trials, and different components/fractions were fixated in only 9.35% of trials. Again, the distribution of transitions across fraction pairs was similar to the pattern of single-digit fractions, only more pronounced (see Figure 4B). NN transitions occurred most frequently for SD pairs (47.33%), while DD transitions were more frequent for the remaining fraction pairs, and transitions occurred more often for the SD and CO fractions pairs. This pattern resulted in a main effect of fraction pair, *F*(3, 57) = 10.60, *p* < .001, 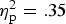, and an interaction of Fraction Pair × Final Transition, *F*(3, 57) = 19.03, *p* < .001, 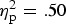. The main effect of final transition was not significant, *F*(1, 19) < 1.

### Discussion

Fraction comparison can be simplified by the use of component-based strategies—that is, by comparing numerators and denominators. In the present experiment, we analysed eye movement sequences to explore the use of different component-based strategies in fraction comparison. Our results show that component-based strategies play an important role in fraction comparison. For the case that the type of fraction pair at hand is unknown, we showed that fraction comparison proceeds with an initial exploratory phase followed by a comparison phase. As expected, we found that eye movements became more specific for the type of fraction pair presented over time. This indicates that participants adapted their processing of fractions flexibly during the task. Finally, the choice of the scan path for the first four exploratory fixations already determined response times: We found that denominator–denominator transitions speeded up the comparison task, indicating that denominators are more important in fraction comparison. Taken together, our results demonstrate how eye movements reflect and shape strategies employed in fraction comparison.

When asked, participants often state to use component-based strategies when confronted with fraction pairs that have common components (Faulkenberry & Pierce, 2011). Depending on the type of fraction pair presented, however, the application of component-based strategies can be more or less difficult. When denominators are the same (SD fraction pairs), only the numerators have to be compared, with the greater numerator giving the greater fraction. This is easier than in the case of SN pairs, because in SN pairs the fraction with the smaller denominator is the greater fraction. When denominators and numerators differ, it is more difficult but often still possible to use a component-based strategy, at least for the CO pairs. Consistent with previous results (Huber et al., 2014; Ischebeck et al., 2009), SD pairs were easiest, producing the fastest RTs and the lowest error rates, followed by the same numerator (SN) fraction pairs. The congruent (CO) pairs were more difficult. The longest RTs and most errors were observed for the incongruent (IC) pairs where a component-based strategy can no longer be used. Furthermore, a partial distance effect was observed. Taken together, these results are compatible with a predominant use of component-based strategies in fraction comparison. Similar results were obtained by the analysis of the number of fixations, which paralleled the RT results. This result confirms the sensitivity of these measures to task difficulty (Williams et al., 1997).

Our main focus of analysis, however, was to explore how eye movements shape and reflect fraction comparison strategies. We analysed the initial four fixations, scan path triplets following the first four fixations, and the final two fixations. We found that viewing patterns became more specific during the task, indicating that participants adapted their eye movements flexibly. As we had taken care in our experiment that each of the fractions’ components had to be fixated in order to be identified, we had hypothesized that the first four fixations mainly served to determine the type of fraction pair presented. We found that viewing patterns were very stereotypical, with two specific patterns accounting for approximately half of all scan paths. Both viewing patterns started with the left fraction and proceeded to the right. It is possible that this is in part influenced by the left-to-right reading direction of our participants (native speakers of German). Reading direction has been found to influence some aspects of number processing, such as the spatial representation of the mental number line (Dehaene, Bossini, & Giraux, 1993).

Additionally, we found that initial fixation sequences that contained a denominator–denominator transition speeded up response times regardless of the type of fraction pair presented. This indicates that the first four fixations did not only serve to establish the type of fraction pair. Our results indicate that inspecting the two denominators in close succession constitutes an advantage in fraction processing. This might be due to denominators playing a more prominent role in fraction comparison than numerators. Although both values are necessary to correctly assess the value of a fraction, the denominator of a fraction is inversely related to the overall value of the fraction. Huber et al. (2014) observed more fixations on the denominators than on the numerators. The authors interpreted this as evidence for the greater processing difficulty associated with denominators.

After analysing the first four fixations, our analysis focused on the triplets following those initial fixations. These triplets were found to depend on the fraction type presented, indicating that eye movements reflected the specific strategy chosen by the participant. In SD fraction pairs, we found that triplets, which consist of numerator fixations that alternate between the two fractions (NNN), occurred predominantly. Similarly, when a SN fraction pair was compared, more DDD triplets were observed. No distinct pattern in the eye movement sequences, however, was observed for CO and IC trials. This could indicate that both types of fraction pairs were processed similarly rather than differently. It is possible that participants realized during the initial exploratory phase that a component-based strategy is less productive in these cases and rather estimated the numerical value of the two fractions. This interpretation is supported by the numerical distance effect observed for CO and IC fraction pairs. Overall, our results show that eye movements flexibly accompany and reflect the comparison strategy chosen by the participant.

Lastly, we analysed the final eye movement sequence consisting of two fixations before the manual response. Overall, the pattern of eye movements was very systematic. In approximately 90% of the trials, either the same component of either fraction was fixated (NN, DD) or the components of the same fraction were fixated (LL, RR). Different patterns emerged for the different fraction pairs. An NN transition was most frequent in SD trials. In all other trials, DD transitions predominated, again confirming the important role of the denominators in skilled fraction comparison.

Comparing the results for the initial four fixations and the final two fixations suggests that eye movements became more and more systematic as the trial proceeded. Whereas the initial four fixations followed rather stereotypical viewing patterns, later fixations reflected the strategy choice for the most efficient comparison of components, especially in the SN and SD trials. In the last two fixations, there were only about 10% of fixation sequences that did not reflect a direct comparison of the same components of either fraction or a comparison of numerator and denominator of the same fraction. This emphasizes that eye movements are more and more driven by strategy choice as the trial proceeds.

Our results for double-digit fractions closely paralleled the results of the single-digit fractions. Participants’ performance hardly differed between single- and double-digit fractions with respect to error rates, response times, and number of fixations (see Table 3). This is surprising since one would assume that double-digit fractions are more difficult to compare (see Table 1). We also found the same basic differences in eye movement sequences with respect to the different fraction pairs. These differences were even more pronounced for double-digit fractions than for single-digit fractions (see Figures 2 [Fig F0003]–4). The similar difficulty of two-digit fraction pairs might be due to participants working on double-digit fractions always after having worked on single-digit fractions. An alternative interpretation would propose that participants compensated for the greater difficulty of double-digit problems by applying strategic eye movements even more rigorously, thus allowing them to solve these problems at the same performance level as the single-digit problems. In any case, our results demonstrate that basic findings (response times, error rates, and number of fixations) as well as complex eye movement patterns can be generalized from the domain of single-digit fractions to double-digit fractions.

Our results confirm and expand the results by Huber et al. (2014). Huber et al. were the first to use eye movements in fraction comparison. They used single-digit fractions (SD, SN, and mixed pairs), which they presented either blocked or in random order. Similar to their study, we found that the number of fixations closely matched response times for different fraction pairs, indicating that participants used component-based strategies most of the time. Different from their study, we evaluated the additional information contained in participants’ scan paths. We found that initial, middle, and final sequences of eye movements reveal the temporal organization of fraction comparison. We found that participants’ eye movement patterns became more indicative for the specific comparison strategy employed over the course of a trial. The specificity of these eye movements also shows that participants were able to adapt their behaviour flexibly to the fraction pair at hand. Our finding of different phases in the viewing patterns for a fraction comparison problem and the reflection of comparison strategies in eye movement sequences is new and expands the findings by Huber et al. Furthermore, we were able to show that the pattern of eye movements in the exploratory phase also shapes response times, as trials with an initial denominator–denominator transition were responded to faster. This is compatible with but goes beyond the results of Huber et al., who found that denominators were fixated overall more often than numerators.

To conclude, the measurement of eye movements in their temporal order is a powerful tool when investigating the adaptive behaviour of participants during a trial. Eye movement sequences provide valuable information above and beyond response times and error rates. Specifically, we were able to show how closely eye movements are related to the use of a specific strategy. Different eye movement sequences predominated depending on the fraction comparison pair presented based on the most effective component-based strategy. We also found that participants showed a remarkable flexibility with regard to strategy use. On a trial-by-trial basis they were able to adjust to the type of fraction pair presented and to adapt their eye movements accordingly. Our results support the use of eye movement measurements in the exploration of strategic adaptation in complex tasks.
